# Tiling and weak tiling in $(\mathbb {Z}_p)^d$

**DOI:** 10.1007/s43670-023-00073-7

**Published:** 2023-12-04

**Authors:** Gergely Kiss, Dávid Matolcsi, Máté Matolcsi, Gábor Somlai

**Affiliations:** 1 Alfréd Rényi Institute of Mathematics, HUN-REN, Reáltanoda u. 13-15, 1053 Budapest, Hungary; 2 Eötvös Loránd University, Pázmány Péter sétány 1/C, 1111 Budapest, Hungary; 3 Department of Analysis, Institute of Mathematics, Budapest University of Technology and Economics (BME), Müegyetem rkp. 3, 1111 Budapest, Hungary

**Keywords:** Fuglede’s conjecture, Translational tiles, Spectral sets, Weak tiling, Abelian p-groups, 20K01, 43A25, 22B05

## Abstract

We discuss the relation of tiling, weak tiling and spectral sets in finite abelian groups. In particular, in elementary *p*-groups $(\mathbb {Z}_p)^d$, we introduce an averaging procedure that leads to a natural object of study: a 4-tuple of functions which can be regarded as a common generalization of tiles and spectral sets. We characterize such 4-tuples for $d=1, 2$, and prove some partial results for $d=3$.

## Introduction

The concept of weak tiling was introduced recently in [15], in connection with Fuglede’s conjecture for convex bodies in $\mathbb {R}^d$. In this note we will study the relation of tiling, weak tiling and spectral sets in finite abelian groups, with particular attention to elementary *p*-groups $(\mathbb {Z}_p)^d$. Our motivation is to give a new tool to prove the “spectral $\rightarrow$ tile” direction of Fuglede’s conjecture in finite abelian groups.

We begin by recalling the necessary notions and fixing our notation.

The cardinality of any finite set *A* will be denoted by |*A*|. We use the standard notation $A\pm B=\{a\pm b: a\in A, \ b\in B\}$, and $kA=\{ka: \ a\in A\}$, for any positive integer *k*. For any *n*, let $\mathbb {Z}_n$ denote the cyclic group of order *n*.

Let *G* be a finite abelian group. A *character* is a homomorphism from *G* to the complex unit circle $\mathbb {T}$. The dual group, denoted by ${\widehat{G}}$, is the collection of all characters of *G*. For a function $f: G\rightarrow \mathbb {C}$, the Fourier transform ${\widehat{f}}: {\widehat{G}}\rightarrow \mathbb {C}$ is defined as

$$\begin{aligned} {\widehat{f}}(\gamma )=\sum _{x\in G} f(x)\gamma (x). \end{aligned}$$

A set $A\subset G$ is called *spectral* if the function space $L^2(A)$ admits an orthogonal basis consisting of characters restricted to *A*. Such an orthogonal basis of characters is called a *spectrum* of *A*. (The spectrum, if it exists, is not necessarily unique.)

We say that a set $A\subset G$ *tiles* *G* if there exists another set $B\subset G$ such that each $g\in G$ can be written uniquely as $g=a+b$, where $a\in A, b\in B$. We usually express this relation as $A\oplus B=G$ or, in the functional notation, $1_A*1_B=1_G$, where $*$ denotes convolution. The convolution of any two functions $f, g: G\rightarrow \mathbb {C}$ is defined in the standard way as $(f*g) (x)=\sum _{y\in G} f(x-y)g(y)$.

For any function $f:G\rightarrow \mathbb {C}$ the function $f_{-}$ is defined as $f_{-}(x)=f(-x)$. Some basic properties of the Fourier transform read as follows: $\widehat{f*g}={\widehat{f}}\cdot {\widehat{g}}$, $\widehat{f_{-}}(\gamma )=\overline{{\widehat{f}}{(\gamma )}}$, $\widehat{f*f_{-}}=|{\widehat{f}}|^2$, and for real-valued even functions $\widehat{{\widehat{f}}}=|G|f$.

Fuglede’s conjecture [7] stated that a set $A\subset G$ is spectral if and only if it tiles *G*. The conjecture was formulated explicitly in $\mathbb {R}^d$, but Fuglede already mentioned that the notions make sense in any locally compact abelian group *G*. In fact, the first counterexample by Tao [20] in $\mathbb {R}^5$ was based on a counterexample in the finite group $(\mathbb {Z}_3)^5$. Since then, further counterexamples [5, 12] have been constructed (all based on examples in finite groups) to both directions of the conjecture in $\mathbb {R}^d$, $d\ge 3$. The conjecture (both directions of it) remains open for $\mathbb {R}$ and $\mathbb {R}^2$. Some further connections between the discrete and the continuous settings have been revealed by Dutkay and Lai in [2].

In this paper we will restrict our attention to finite abelian groups, and mostly to the case $G=(\mathbb {Z}_p)^d$ with *p* being a prime. For finite abelian groups, several results have been discovered in recent years [3, 4, 6, 8–11, 13, 14, 16–19, 21]. For the purposes of this note, we single out the results of [8] and [6, 17]. In [8] the authors prove both directions of Fuglede’s conjecture in $(\mathbb {Z}_p)^2$. To the contrary, in [6] and [17] the authors prove (independently of each other) that for odd primes *p* there exist spectral sets in $(\mathbb {Z}_p)^d$, $d\ge 4$, which do not tile the group, thus exhibiting a counterexample to the “spectral $\rightarrow$ tile” direction of Fuglede’s conjecture in these groups.

In connection with Fuglede’s conjecture, the notion of *weak tiling* was introduced in [15]. For us, in the case of finite abelian groups, weak tiling can be formulated as follows. A set $A\subset G$ tiles another set $E\subset G$ *weakly*, if there exists a nonnegative function $h: G\rightarrow \mathbb {R}$ such that $1_A*h=1_E$. We will soon see that it makes sense to introduce the further restrictions that $h(0)=1$, the function *h* is positive definite (i.e. ${\widehat{h}}\ge 0$), and restrict our attention to the case $E=G$.

### Definition 1.1

We say that a set $A\subset G$ *pd-tiles* *G* *weakly* if there exists a nonnegative function $h: G\rightarrow \mathbb {R}$ such that $h(0)=1$, $1_A*h=1_G$, and ${\widehat{h}}\ge 0$.

In the terminology, ”pd” stands for positive definite. Note that, due to positive definiteness, *h* is necessarily an even function, $h(x)=h(-x)$. The assumption $h(0)=1$ is essential, otherwise the constant function $h=\frac{1}{|A|}$ would provide a weak pd-tiling for any set $A\subset G$. Note also, as a comparison with the terminology in [15], that if *A* pd-tiles *G* weakly then *A* tiles its complement weakly.

A simple but important observation, also essentially contained in [15], is that if *A* pd-tiles *G* weakly with a function *h*, then

1 $$\begin{aligned} A-A \ \cap \ \textrm{supp}\ h=\{0\}. \end{aligned}$$

The reason is that $x=a-a'$ and $h(x)>0$ would imply $1_A*h(a)\ge h(0)1_A(a)+h(x)1_A(a')=1+h(x)>1$.

Next, we show that a proper tiling always induces a weak pd-tiling of *G*.

### Lemma 1.2

If $A\oplus B=G$ is a tiling, then *A* pd-tiles *G* weakly with $h_1=\frac{1}{|B|} 1_B*1_{-B}$.

### Proof

$A\oplus B=G$ implies ${\widehat{1}}_A \cdot {\widehat{1}}_B = |G|\delta _0$, so that the supports of the functions ${\widehat{1}}_A$ and ${\widehat{1}}_B$ are essentially disjoint (only intersect at 0). This implies that ${\widehat{1}}_A \cdot \frac{1}{|B|}|{\widehat{1}}_B|^2 = |G|\delta _0$, which, in turn, implies $1_A*\left( \frac{1}{|B|}1_B*1_{-B} \right) =1_G$. $\square$

We remark that the notation $h_1$ above (instead of using simply *h*) is for later convenience.

The essential observation in [15] is that if a set *A* is spectral, then it tiles its complement weakly. However, slightly more is true.

### Lemma 1.3

Let *G* be a finite abelian group. If $A\subset G$ is spectral, then *A* pd-tiles *G* weakly.

### Proof

Let $S\subset {\widehat{G}}$ be a spectrum of *A*. Then $|S|=|A|$ because the space $L^2(A)$ has dimension |*A*|, and *S* is a basis. Let $h_1=\frac{1}{|A|^2}|{\widehat{1}}_S|^2$. Then $h_1\ge 0$, $h_1(0)=1$, ${\widehat{h}}_1=\frac{|G|}{|A|^2}1_S*1_{-S}\ge 0$, so $h_1$ is nonnegative, normalized and positive definite, as required. Also, the weak tiling condition $1_A*h_1=1_G$ is most easily seen by taking Fourier transforms. ${\widehat{1}}_A \cdot \left( \frac{|G|}{|A|^2} 1_S*1_{-S} \right) =|G|\delta (0)$ is true because ${\widehat{1}}_A(0)=|A|$, $1_S*1_{-S}(0)=|S|=|A|$, and the support of $1_S*1_{-S}$ is $S-S$, which is a subset of $\{{\widehat{1}}_A=0\}\cup \{0\}$ by the orthogonality of *S* in $L^2(A)$. $\square$

By this lemma, we can establish the “spectral $\rightarrow$ tile” direction of Fuglede’s conjecture in a finite abelian group *G*, if we prove that any set that pd-tiles *G* weakly, actually tiles *G* properly. This motivates the following definition.

### Definition 1.4

Assume that a finite abelian group *G* has the property that whenever a set *A* pd-tiles *G* weakly then *A* tiles *G* properly. Then we call the group *G* *pd-flat*.

In the remainder of this note we will focus our attention on elementary *p*-groups $G=(\mathbb {Z}_p)^d$. As mentioned above, for odd primes and $d\ge 4$, there exist spectral sets in *G* which do not tile *G*. Therefore, $(\mathbb {Z}_p)^d$ is not pd-flat for $d\ge 4$.

In Sect. 2 we introduce an averaging technique which leads to a natural generalization of spectral sets and tiles in $(\mathbb {Z}_p)^d$. Finally, in Sect. 3 we show that $(\mathbb {Z}_p)^d$ is pd-flat for $d=1, 2$, and we give some partial results and conjectures for $d=3$.

## Averaging

It is natural to go one step further with the generalization of tiling.

### Definition 2.1

Let $f, h: G\rightarrow \mathbb {R}$ be nonnegative functions such that $f(0)=h(0)=1$, ${\widehat{f}}\ge 0, {\widehat{h}}\ge 0$. We say that the pair (*f*, *h*) is a *functional pd-tiling* of *G* if $f*h=1_G$.

It turns out that this notion is very flexible, and for $G=(\mathbb {Z}_p)^d$ it gives rise to a natural averaging procedure. In connection with this, we introduce a special class of functions.

### Definition 2.2

We say that a function $f: (\mathbb {Z}_p)^d\rightarrow \mathbb {C}$ is *ray-type*, if for any $\textbf{x}\in (\mathbb {Z}_p)^d$, and any $k=1, \dots , p-1$ we have $f(k\textbf{x})=f(\textbf{x})$.

That is, a ray-type function is constant on any punctured line through the origin (but may have a different value at the origin).

We also need some information on the zeroes of the Fourier transform of the indicator function $1_A$ of a set $A\subset (\mathbb {Z}_p)^d$. We make the identification $G=\{0, 1, \dots , p-1\}^d$, and the same for the dual group ${\widehat{G}}=\{0, 1, \dots , p-1\}^d$. It is sometimes useful to think of the elements of *G* as *column* vectors of length *d*, and elements of ${\widehat{G}}$ as *row* vectors of length *d*. Also, in notation, we will use boldface letters for elements of *G* and ${\widehat{G}}$ to indicate that they are vectors. With these identifications in mind, the action of a character $\textbf{t}\in {\widehat{G}}$ on an element $\textbf{x}\in G$ is given by $e^{2i\pi \langle \textbf{t}, \textbf{x}\rangle /p}$.

For a function $f: G\rightarrow \mathbb {C}$, and $\textbf{t}\in {\widehat{G}}$, we have ${\widehat{f}} (\textbf{t})=\sum _{\textbf{x}\in G} f(\textbf{x}) e^{2i\pi \langle \textbf{t}, \textbf{x}\rangle /p}$. In particular, for $A\subset G$, the Fourier transform of $1_A$ takes the form ${\widehat{1}}_A(\textbf{t})=\sum _{\textbf{a}\in A} e^{2i\pi \langle \textbf{t}, \textbf{a}\rangle /p}$.

The important point here is that for any $\textbf{t}\ne 0$ we either have ${\widehat{1}}_A(k\textbf{t})=0$ for all $k=1, 2, \dots , p-1$, or ${\widehat{1}}_A(k\textbf{t})\ne 0$ for all $k=1, 2, \dots , p-1$. This is well-known, and the reason is that ${\widehat{1}}_A(\textbf{t})=0$ if and only if the sum $\sum _{\textbf{a}\in A} e^{2i\pi \langle \textbf{t}, \textbf{a}\rangle /p}$ contains the same number of terms for each *p*th root of unity, in which case $\sum _{\textbf{a}\in A} e^{2i\pi \langle k\textbf{t}, \textbf{a}\rangle /p}$ also contains the same number of terms of each *p*th root of unity.

Therefore, the zeros of the Fourier transform ${\widehat{1}}_A$ consist of punctured lines through the origin (for $\textbf{t}\ne 0$ a punctured line ${\dot{L}}$ is given by ${\dot{L}}=\{\textbf{t}, 2\textbf{t}, \dots , (p-1)\textbf{t}\}$; note that $0\notin {\dot{L}}$, and we use the notation $L={\dot{L}}\cup \{0\}$). The same is true for the zeroes of the Fourier transform of $f=\frac{1}{|A|} 1_A*1_{-A}$, because ${\widehat{f}}=\frac{1}{|A|}|{\widehat{1}}_A|^2$.

We can now perform the first half of the averaging.

### Lemma 2.3

Let $G=(\mathbb {Z}_p)^d$, and $1_A*h_1=1_G$ be a weak pd-tiling of *G* by a set *A*. Then, for any $k=1, \dots , p-1$, $1_{kA}*h_1=1_G$ is also a weak pd-tiling. Furthermore, with the notation $f_k=\frac{1}{|A|}1_{kA}*1_{-kA}$, we have that $f_k*h_1=1_G$ is a functional pd-tiling. Finally, for $f=\frac{1}{p-1}\sum _{k=1}^{p-1} f_k$ we have that *f* is a ray-type function, $\textrm{supp}\ f \ \cap \ \textrm{supp}\ h_1=\{0\}$, and $f*h_1$ is also a functional pd-tiling.

### Proof

Note that ${\widehat{1}}_{kA}(\textbf{t})={\widehat{1}}_A(k\textbf{t})$, so the zeroes of these functions are the same punctured lines through the origin.

The assumption $1_A*h_1=1_G$ is equivalent to ${\widehat{1}}_A {\widehat{h}}_1=|G|\delta _0$. Due to the fact that the zeroes of ${\widehat{1}}_A$ and ${\widehat{1}}_{kA}$ coincide, and $|A|=|kA|$, we have ${\widehat{1}}_{kA} {\widehat{h}}_1=|G|\delta _0$, and therefore $1_{kA}*h_1=1_G$ is a weak pd-tiling.

The function $f_k=\frac{1}{|A|}1_{kA}*1_{-kA}$ is nonnegative, $f_k(0)=1$, ${\widehat{f}}_k=\frac{1}{|A|}|{\widehat{1}}_{kA}|^2\ge 0$, and the zeroes of ${\widehat{f}}_k$ and ${\widehat{1}}_{kA}$ coincide. Also, ${\widehat{f}}_k(0)=|A|={\widehat{1}}_{kA}(0)$, therefore ${\widehat{f}}_k {\widehat{h}}_1=|G|\delta _0$, which implies that $f_k*h_1$ is a functional pd-tiling. Also, $\textrm{supp}\ f_k=kA-kA$, and hence $\textrm{supp}\ f_k \ \cap \ \textrm{supp}\ h_1=\{0\}$ by (1).

Finally, for the average $f=\frac{1}{p-1}\sum _{k=1}^{p-1} f_k$, we clearly have $f\ge ,0$, ${\widehat{f}}\ge 0$, $f(0)=1$ and $f*h_1=|G|\delta _{0}$ is a functional pd-tiling since these properties hold for each $f_i$. Also, by averaging, it is clear that *f* is ray-type, and the property $\textrm{supp}\ f \ \cap \ \textrm{supp}\ h_1=\{0\}$ is inherited. $\square$

We can perform a second step of averaging for the function $h_1$.

### Lemma 2.4

Let $G=(\mathbb {Z}_p)^d$, and $1_A*h_1=1_G$ be a weak pd-tiling of *G* by a set *A*, and define the ray-type function *f* as in Lemma 2.3. For any $k=1, \dots , p-1$, let $h_k(\textbf{x})=h_1(k\textbf{x})$, and let $h=\frac{1}{p-1}\sum _{k=1}^{p-1}h_k$. Then, for each *k*, $f*h_k=1_G$ is a functional pd-tiling, the average *h* is a ray-type function, and $f*h$ is also a functional pd-tiling such that $\textrm{supp}\ f \ \cap \ \textrm{supp}\ h=\{0\}$.

### Proof

Recall from Lemma 2.3 that *f* is a ray-type function. We claim that ${\widehat{f}}$ is also ray-type. To see this, note that *f* can be written in the form $f=c\delta _0+\sum _{i} c_i1_{L_i}$ for some lines $L_i$ (for convenience, we use proper lines in this decomposition, not punctured lines; the difference is absorbed in the constant *c* at the origin). The Fourier transform of $1_{L_i}$ is $p1_{H_i}$ with the hyperplane $H_i$ being orthogonal to $L_i$, and therefore the ${\widehat{f}}=c1_{{\widehat{G}}}+p\sum _i 1_{H_i}$, which is clearly ray-type.

By Lemma 2.3 we have that $f*h_1=1_{G}$, which implies ${\widehat{f}} {\widehat{h}}_1=|G|\delta _0$. This means that $\textrm{supp}\ {\widehat{f}}\cap \textrm{supp}\ {\widehat{h}}_1=\{0\}$. As ${\widehat{f}}$ is ray-type, its support is a union of lines $R_i$, and hence ${\widehat{h}}_1$ must be 0 on the punctured lines $\dot{R_i}$. But ${\widehat{h}}_k(\textbf{t})={\widehat{h}}_1(\textbf{t}/k)$, so ${\widehat{h}}_k$ is also 0 on ${\dot{R}}_i$, and hence ${\widehat{f}} {\widehat{h}}_k=|G|\delta _0$ holds (for the value at zero we have ${\widehat{h}}_1(0)={\widehat{h}}_k(0)=|G|/|A|$). In turn, this implies that $f*h_k=1_G$ is indeed a functional pd-tiling.

Also, $\textrm{supp}\ f \ \cap \ \textrm{supp}\ h_1=\{0\}$ by Lemma 2.3, and *f* is ray-type, so the support of $h_1$ is contained in rays where $f=0$. This clearly implies that the support of $h_k$ is also contained in these rays, and therefore $\textrm{supp}\ f \ \cap \ \textrm{supp}\ h_k=\{0\}$

After averaging, it is clear that *h* is ray-type, and the functional pd-tiling property $f*h$ is preserved, as well as the condition $\textrm{supp}\ f \ \cap \ \textrm{supp}\ h=\{0\}$. $\square$

We see from Lemmas 1.2 and 1.3 that *A* pd-tiles *G* weakly in both cases when *A* tiles *G*, or *A* is spectral in *G*. After applying Lemmas 2.3 and 2.4 we arrive at the following corollary.

### Corollary 2.5

Let $G=(\mathbb {Z}_p)^d$, and assume *A* pd-tiles *G* weakly with $1_A*h_1=1_G$ (in particular, this is the case if *A* tiles *G*, or *A* is spectral in *G*). For $k=1, \dots , p-1$, let $f_k=\frac{1}{|A|}1_{kA}*1_{-kA}$, $h_k(\textbf{x})=h_1(k\textbf{x})$, and $f=\frac{1}{p-1}\sum _{k=1}^{p-1}f_k$, $h=\frac{1}{p-1}\sum _{k=1}^{p-1}h_k$. Then

2 $$\begin{aligned} |A|=\sum _{x\in G}f(x), \end{aligned}$$

and the 4-tuple of functions $(f, h, {\widehat{f}}, {\widehat{h}})$ satisfy the following properties:

(i) $f, h, {\widehat{f}}, {\widehat{h}}$ are all ray-type functions,
(ii) $$f\ge 0$$, $$h\ge 0$$,
(iii) $$f(0)=1$$, $$h(0)=1$$,
(iv) $$f*h=1_G$$,
(v) $$\textrm{supp}\ f \cap \textrm{supp}\ h=\{0\}$$,
(vi) $${\widehat{f}}\ge 0$$, $${\widehat{h}}\ge 0$$,
(vii) $${\widehat{f}}(0)=|A|, \ {\widehat{h}}(0)=|G|/|A|$$,
(viii) $${\widehat{f}}*{\widehat{h}}=|G|1_{{\widehat{G}}}$$,
(ix) $$\textrm{supp}\ {\widehat{f}} \cap \textrm{supp}\ {\widehat{h}}=\{0\}$$.

### Proof

Only (viii) needs further explanation, and it follows by taking Fourier transform of the equation, noting that $\widehat{{\widehat{f}}}=|G|f$ (and the same for *h*), and applying (v) and (iii). $\square$

This corollary motivates the following definition.

### Definition 2.6

A 4-tuple of functions $(f, h, {\widehat{f}}, {\widehat{h}})$ is called a *complementary 4-tuple* if they satisfy conditions (i)-(ix) of Corollary 2.5. (We do not necessarily require that *f* be constructed from a set *A*.)

The notion of complementary 4-tuples is very appealing, because it puts the functions *f*, *h* and their Fourier transform ${\widehat{f}}, {\widehat{h}}$ on equal footing. When studying spectral sets and tiles in $(\mathbb {Z}_p)^d$ it is natural to try to characterize all complementary 4-tuples. We will do this for $d=1, 2$ in the next section, and present some partial results for $d=3$.

We also remark that an analogous averaging procedure can be carried out in cyclic groups $\mathbb {Z}_n$, leading to a similar notion of complementary 4-tuples in those groups. Studying these 4-tuples could lead to some insights concerning the Coven-Meyerowitz conjecture. This is subject to future research.

## Complementary 4-tuples in $(\mathbb {Z}_p)^d$

In this section we analyze complementary 4-tuples in $(\mathbb {Z}_p)^d$, and prove that $(\mathbb {Z}_p)^d$ is pd-flat for $d=1,2$, and also give some partial results for $d=3$.

For convenience, we recall here what is known about Fuglede’s conjecture in these groups. Both directions of the conjecture are true for $d=1, 2$, with $d=1$ being fairly trivial, and $d=2$ being treated in [8]. A simpler proof of the case $d=2$ can be found in [10]. For $d=3$ it is known that all tiles are spectral [1], but it is not known whether all spectral sets are tiles. For $d\ge 4$ (and *p* being odd) there are examples of spectral sets which do not tile the group [6, 17] but the tile-spectral direction of the conjecture is open.

We now turn to the main results of this section. We emphasize here that a group *G* being pd-flat is *formally* stronger than the “spectral $\rightarrow$ tile” direction of Fuglede’s conjecture in *G*.

### Proposition 3.1

$G=(\mathbb {Z}_p)^d$ is pd-flat for $d=1, 2$, that is, every set *A* which pd-tiles *G* weakly, tiles *G* properly. Also, *G* is not pd-flat for $d\ge 4$.

### Proof

Assume *A* pd-tiles *G* weakly. Then there exists a complementary 4-tuple $(f, h, {\widehat{f}}, {\widehat{h}})$ with the properties listed in Corollary 2.5, and *f* is constructed from $1_A$ as in Corollary 2.5.

For $d=1$ the support of any ray-type function is either $\{0\}$ or *G*. If $\textrm{supp}\ f=\{0\}$ then $|A|=1$, and *A* tiles *G* trivially. If $\textrm{supp}\ f=G$ then necessarily $\textrm{supp}\ h=\{0\}$, and $h(0)=1$ implies $h=\delta _0$, and hence *f* must be the constant function 1. Therefore, $|A|=p$ by (2), and hence $A=G$.

For $d=2$ we must perform a case-by-case analysis of the function *f*. Let $c=\inf _{x\in G} f(x)\ge 0$, and let $L_i$ be the lines in the support of *f*. Then *f* can be decomposed uniquely as $f=c1_G+\sum _i d_i1_{L_i}+ m\delta _0$ with some $d_i\ge 0$ and $m\in \mathbb {R}$ (note that $L_i$ denotes the full line here, not the punctured line).

If $c>0$ then $\textrm{supp}\ f=G$, and hence $\textrm{supp}\ h=\{0\}$, $h=\delta _0$, so $f=1_G$. By (2) we obtain that $|A|=p^2$ and $A=G$.

If $c=0$ and $m>0$ then ${\widehat{f}}>0$ everywhere on ${\widehat{G}}$, so $\textrm{supp}\ {\widehat{h}}=\{0\}$. Thus *h* is constant, and $h(0)=1$ implies that *h* is constant 1. This implies that $f=\delta _0$, and $|A|=1$.

If $c=0$ and $m=0$, then the total mass of *f* is exactly *p* times the mass at 0, because this is true for all $1_{L_i}$. The mass at zero is $f(0)=1$, and hence we have $|A|=p$, by equation (2). Also, $c=0$ implies that *f* must be zero on some punctured line ${\dot{R}}$, so *A* can have at most one element in each coset of the line *R* since $A-A \subset \textrm{supp}\ (f)$. But $|A|=p$, so *A* must have exactly one element in each coset of *R*, and therefore $A\oplus R =G$ is a tiling.

Finally, we claim that $c=0$ and $m<0$ is not possible. Indeed, $c=0$ means that *f* must be zero on some punctured line ${\dot{R}}$, and therefore ${\widehat{f}}=m<0$ on the orthogonal punctured line ${\dot{R}}^\perp$, contradicting the nonnegativity of ${\widehat{f}}$.

For $d\ge 4$ we know that there exist spectral sets in $(\mathbb {Z}_p)^d$ which do not tile the group [6, 17]. Therefore, $(\mathbb {Z}_p)^d$ cannot be pd-flat. $\square$

For $d=3$, we conjecture that $(\mathbb {Z}_p)^3$ is pd-flat, but unfortunately we cannot give a complete characterization of complementary 4-tuples, we can only prove some partial results in this direction. Therefore, the “spectral $\rightarrow$ tile” direction of Fuglede’s conjecture remains open in this case. We have the following partial result.

### Proposition 3.2

Let $G=(\mathbb {Z}_p)^3$, and assume $A\subset G$ pd-tiles *G* weakly with $1_A*h_1=1_G$. Let $(f, h, {\widehat{f}}, {\widehat{h}})$ be the complementary 4-tuple constructed in Corollary 2.5. Then either *A* tiles *G* properly, or the functions $f, h, {\widehat{f}}, {\widehat{h}}$ all have the following “dispersive” property: for any 2-dimensional subspaces $S\subset G$, $S'\subset {\widehat{G}}$ the intersections $\textrm{supp}\ f \cap S$, $\textrm{supp}\ h \cap S$, $\textrm{supp}\ {\widehat{f}}\cap S'$ and $\textrm{supp}\ {\widehat{h}} \cap S'$ are all non-trivial (i.e. not equal to $\{0\}$ or the whole plane).

### Proof

Exploiting the fact that all appearing functions are ray-type, we can represent each function in the following normalized form. Let us label the planes and lines through the origin as $S_1, S_2, \dots , S_{p^2+p+1}$ and $L_1, L_2, \dots , L_{p^2+p+1}$. Then *f* can be represented uniquely as

3 $$\begin{aligned} f=w 1_G+ \sum _i (c_i1_{S_i}+d_i1_{L_i})+m\delta _0, \end{aligned}$$

where the coefficients $w, c_i, d_i\ge 0$, $m\in \mathbb {R}$ are defined by the following greedy algorithm: *w* is the maximal value such that $f-w1_G\ge 0$, $c_1$ is the maximal value such that $f- w1_G-c_1 1_{S_1}\ge 0$, $c_2$ is the maximal value such that $f-w1_G-c_11_{S_1}-c_21_{S_2}\ge 0$, etc, and subsequently $d_1$ is the maximal value such that $f-w1_G-(\sum _{i=1}^{p^2+p+1} c_i1_{S_i})- d_11_{L_1}\ge 0$, etc. We can represent $f, h, {\widehat{f}}, {\widehat{h}}$ in this manner uniquely, after fixing the order of planes and lines in *G* and ${\widehat{G}}$.

We will show that if any of $f, h, {\widehat{f}}, {\widehat{h}}$ does not have the dispersive property (stated in the proposition), then *A* tiles *G*. This is done by a case-by-case analysis, where we will use the properties of complementary 4-tuples stated in Corollary 2.5.

Case I. Let us first consider the trivial case when the support of some of the appearing functions is the whole underlying group.

(a) If $\textrm{supp}\ f=G$, then $\textrm{supp}\ h=\{0\}$ by (v), and hence $h=\delta _0$ by (iii), and $f=1_G$ by (iv), and $|A|=p^3$ by (2). Therefore, $A=G$, and *A* tiles the group trivially.

(b) Similarly, if $\textrm{supp}\ h=G$ then $\textrm{supp}\ f=\{0\}$, and $|A|=1$, and *A* tiles *G* trivially.

(c) If $\textrm{supp}\ {\widehat{f}}={\widehat{G}}$, then $\textrm{supp}\ {\widehat{h}}=\{0\}$ by (ix), and hence *h* is a constant function, $h=1_G$ by (iii), and therefore $f=\delta _0$ by (iv), and again $|A|=1$.

(d) Similarly, if $\textrm{supp}\ {\widehat{h}}={\widehat{G}}$, then ${\widehat{f}}=\{0\}$, and hence *f* is constant 1, and $|A|=p^3$ by (2).

Having dealt with Case I, we can assume for the rest of the argument that $w=0$ in equation (3). Moreover, this also implies that $m\le 0$ in (3) because $m>0$ would imply $\textrm{supp}\ {\widehat{f}}={\widehat{G}}$. Altogether, we can conclude that the representation of *f* takes the form

4 $$\begin{aligned} f=\sum _i (c_i1_{S_i}+d_i1_{L_i})+m\delta _0, \end{aligned}$$

where $c_i, d_i\ge 0$, and $m\le 0$. The same is true for the representations of $h, {\widehat{f}}, {\widehat{h}}$.

Case II. Next, assume that the support of one of the appearing functions intersects a plane only at 0.

(a) Let $\textrm{supp}\ f \ \cap \ S=\{0\}$. Let ${\dot{L}}\subset {\widehat{G}}$ denote the punctured line orthogonal to *S*. Then ${\widehat{f}}=m\le 0$ on ${\dot{L}}$, and the nonnegativity of ${\widehat{f}}$ implies $m=0$. Also, the “empty” plane *S* intersects every other plane $S_i$, so every $S_i$ has at least one “empty” line $S\cap S_i$, and hence the weight $c_i$ must be 0 in equation (4) for every *i*. Hence, *f* can be represented in the form $f=\sum _i d_i 1_{L_i}$, and therefore the total mass of *f* is *p* times the mass at 0 (as this is the case for every $L_i$), and hence $|A|=p$ is implied by (2) and $f(0)=1$. Furthermore, the fact that *S* is empty means that $A-A \ \cap \ S=\{0\}$, so there is exactly one point of *A* on each coset of *S*. Therefore, $A\oplus S=G$ is a tiling.

(b) If $\textrm{supp}\ h \ \cap \ S=\{0\}$, then the same argument as in (a) shows that *h* has a decomposition $h=\sum _i {\tilde{d}}_i L_i$, and therefore $\frac{\sum _{\textbf{x}\in G} h(\textbf{x})}{h(0)}=p$. Considering $h(0)=1$, this implies $\sum _{\textbf{x}\in G} h(x)=p$, and hence $|A|=p^2$ by (iv). Also, $\textrm{supp}\ f\ne G$, so there exists a line *L* such that $A-A \ \cap \ L=\{0\}$, and hence $A\oplus L=G$ is a tiling.

(c) If $\textrm{supp}\ {\widehat{f}} \ \cap \ S'=\{0\}$, then the same argument as in (a) shows that ${\widehat{f}}$ has a decomposition ${\widehat{f}}=\sum _i d'_i L'_i$, and therefore $\frac{\sum _{\textbf{t}\in {\widehat{G}}} {\widehat{f}}(t)}{{\widehat{f}}(0)}=p$. Considering that ${\widehat{f}}(0)=\sum _{\textbf{x}\in G}f(x)$ and $\sum _{\textbf{t}\in {\widehat{G}}} {\widehat{f}}(t)=p^2 f(0)$, we obtain $\sum _{\textbf{x}\in G}f(\textbf{x})=p^2$, that is $|A|=p^2$ by (2). We can finish the argument as in (b): as $\textrm{supp}\ f\ne G$, there exists a line *L* such that $A-A \ \cap \ L=\{0\}$, and hence $A\oplus L=G$ is a tiling.

(d) If $\textrm{supp}\ {\widehat{h}} \ \cap \ S'=\{0\}$, then the same argument as in (a) shows that ${\widehat{h}}$ has a decomposition ${\widehat{h}}=\sum _i {\tilde{d}}'_i L'_i$, and therefore $\frac{\sum _{\textbf{t}\in {\widehat{G}}} {\widehat{h}}(t)}{{\widehat{h}}(0)}=p$. This implies that $\sum _{\textbf{x}\in G}h(\textbf{x})=p^2$, and $\sum _{\textbf{x}\in G}f(\textbf{x})=p$. This implies that in the decomposition (4) all coefficients $c_i=0$ and $m=0$, otherwise the total mass of *f* would be greater than *p* times the mass at 0 (recall that $c_i\ge$ and $m\le 0$). Therefore, $f=\sum _i d_i 1_{L_i}$. Also, as $\textrm{supp}\ {\widehat{f}}\ne {\widehat{G}}$, we have a line $L'\subset {\widehat{G}}$ such that ${\widehat{f}}=0$ on $\dot{L'}$, which implies that $f=0$ on the punctured plane $S=L'^\perp$. Finally, as $|A|=p$ and $A-A \ \cap \ S=\{0\}$ we have that $A\oplus S=G$ is a tiling.

Case III. Finally, assume that the support of one of the appearing functions contains a whole plane. In this case, we can invoke (v) and (ix) to conclude that the support of some other function intersects the same plane at 0 only, and we are done by Case II above. $\square$

It is worth summarizing here what this result means for spectral sets. For a spectral set $A\subset (\mathbb {Z}_p)^3$, perform the avearaging procedure leading to the complementary 4-tuple in Corollary 2.5. If any of the functions $f, h, {\widehat{f}}, {\widehat{h}}$ does not have the dispersive property described in Proposition 3.2 then *A* necessarily tiles $(\mathbb {Z}_p)^3$. If all appearing functions have the dispersive property, they must have a representation

5 $$\begin{aligned} \sum _i d_i 1_{L_i}+ m\delta _0, \end{aligned}$$

where the lines $L_i$ are situated such that they do not cover any plane fully, and do not leave any plane empty. Furthermore, $d_i> 0$, $m<0$ (the latter is true because $m\ge 0$ would imply the Fourier transform being positive on the planes $L_i^\perp$).

It would be tempting to conjecture that this situation cannot occur, that is, complementary 4-tuples $(f, h, {\widehat{f}}, {\widehat{h}})$ with all functions having the dispersive property do not exist. However, unfortunately, the following example shows that this is not the case.

### Example 3.3

Let $S_1,S_2,S_3$ be three different planes through the origin in $(\mathbb {Z}_p)^3$, and let $L_1=S_2\cap S_3$, $L_2=S_1\cap S_3$, $L_3=S_1\cap S_2$ be the lines of intersection. For the sake of easier calculations we will use a representation for *f* and *h* different from (5). (It is not difficult to re-write the representations below according to equation (5), and we invite the reader to do so.)

Let $$f_0=2 \cdot 1_G- 2(1_{S_1}+1_{S_2}+1_{S_3})+p(1_{L_1}+1_{L_2}+1_{L_3})$$, and $$h_0=(1_{S_1}+1_{S_2}+1_{S_3})- 2(1_{L_1}+1_{L_2}+1_{L_3})+2p\delta _0$$.

Then, $${\widehat{f}}_0= p^2(1_{L_1^\perp }+1_{L_2^\perp }+1_{L_3^\perp })-2p^2(1_{S_1^\perp }+1_{S_2^\perp }+1_{S_3^\perp })+2p^3\delta _0$$, and $${\widehat{h}}_0= 2p1_{{\widehat{G}}}-2p(1_{L_1^\perp }+1_{L_2^\perp }+1_{L_3^\perp })+p^2(1_{S_1^\perp }+1_{S_2^\perp }+1_{S_3^\perp })$$.

Based on these formulae, it is easy (but somewhat tedious) to check that the normalized functions $f=\frac{1}{3p-4}f_0$, $h=\frac{1}{2p-3}{h_0}$, and their Fourier transforms ${\widehat{f}}, {\widehat{h}}$ form a complementary 4-tuplet $(f, h, {\widehat{f}}, {\widehat{h}})$, such that none of the appearing functions have the dispersive property of Proposition 3.2.

It may be easier to visualize this example if we identify the punctured lines ${\dot{L}}_i$ of $(\mathbb {Z}_p)^3$ with points of the projective plane $PG(2,\mathbb {Z}_p)$. In this picture, $S_i$ become lines on the projective plane, and the functions *f* and *h* can be described in terms of a triangle in the projective plane. For example, the function *h* is positive on the lines of a triangle (with the exception of the vertices, where *h* is 0), and zero everywhere outside. We leave the details to the reader.

Notice, however, that in this example neither *f* nor *h* can come from the averaging of an indicator function of a set *A* as in Lemma 2.3. The reason is that by Eq. (2) we would have $|A|=\frac{p^2(2p-3)}{3p-4}$ or $|A|=\frac{p(3p-4)}{2p-3}$, neither of which is an integer for $p\ne 2, 3$, and for $p=2, 3$ it is easy to check (with a finite case-by-case analysis) that *f* and *h* cannot be a result of averaging an indicator function of any set *A*.

Example 3.3 can be generalized: one can take *k* points on the projective plane, with $k-1$ lying on a line, and one point being outside. Then, a complementary 4-tuple $(f, h, {\widehat{f}}, {\widehat{h}})$ can be constructed, such that the function *h* is positive on all the lines connecting these points (with exception of the points themselves, where *h* is 0), and zero everywhere outside. The function *f* is then supported on the complement $\textrm{supp}\ h$. The exact formulae are somewhat cumbersome, so we choose to omit them.

Given that such examples exist, we cannot yet decide whether the “spectral $\rightarrow$ tile” direction of Fuglede’s conjecture holds in these groups.

As a final remark we mention here Rédei’s conjecture which concerns the structure of tilings in $(\mathbb {Z}_p)^3$. It states that in any normalized tiling $A\oplus B =(\mathbb {Z}_p)^3$ (normalized meaning that $0\in A, B$ is assumed) we must have that either *A* or *B* is contained in a proper subgroup.

A complete characterization of complementary 4-tuples in $(\mathbb {Z}_p)^3$ could, in principle, lead to the solution of both Fuglede’s and Rédei’s conjecture in these groups.
